# Cardiac monitoring in memory clinics: national survey of UK practice

**DOI:** 10.1192/bjb.2021.108

**Published:** 2023-02

**Authors:** George Crowther, Noura Ahmed, Deepa Kasa, Zoe Goff, Muzahir H. Tayebjee

**Affiliations:** 1 Leeds and York Partnership NHS Foundation Trust, UK; 2 University of Leeds, UK; 3 Leeds Teaching Hospitals NHS Trust, UK

**Keywords:** Cardiac monitoring, acetylcholinesterase inhibitors, dementia, UK memory clinics, national survey

## Abstract

**Aims and method:**

People diagnosed with dementia are often started on acetylcholinesterase inhibitors (AChEIs). As AChEIs can be associated with cardiac side-effects, an electrocardiogram (ECG) is sometimes requested before treatment. Previous work has suggested there is little consensus as to when or how ECGs should be obtained. This can create inconsistent practice, with patient safety, economic and practical repercussions. We surveyed 305 UK memory clinic practitioners about prescribing practice.

**Results:**

More than 84% of respondents completed a pulse and cardiac history before prescribing AChEIs. Opinion was divided as to who should fund and conduct ECGs. It was believed that obtaining an ECG causes patients inconvenience and delays treatment. Despite regularly interpreting ECGs, 76% of respondents did not update this clinical skill regularly.

**Clinical implications:**

The variation in practice observed has service-level and patient implications and raises potential patient safety concerns. Implementing national guidelines or seeking novel ways of conducting cardiac monitoring could help standardise practice.

In the UK more than 850 000 people live with dementia, a number predicted to rise to over 1.6 million by 2040.^1^ The majority of diagnoses are made in memory clinics that are usually run by mental health service providers and staffed by a range of healthcare professionals, including psychiatrists, mental health nurses, psychologists and occupational therapists.

Donepezil, rivastigmine and galantamine are reversible AChEIs, and are recommended in mild and moderate Alzheimer-type dementia.^2^ In the UK they are most commonly commenced in memory clinics. These drugs have significant central nervous system selectivity, but some peripheral actions can cause cardiac arrhythmias:^3^ most commonly bradycardia secondary to their effect on cardiac conduction,^4–8^ less commonly tachycardia and atrial or ventricular ectopy.^9^ Arrhythmias can predispose to presyncope or syncope. This is important as these drugs are most commonly prescribed to older people, a group who already have a high prevalence of cardiac comorbidity and frailty and are susceptible to falls.

It is perhaps unsurprising therefore that clinicians may be concerned about the pro-arrhythmic potential of AChEIs. Routine practice to screen patients for susceptibility to this usually involves taking a cardiac history (including drugs), checking a radial pulse, blood pressure monitoring or performing an electrocardiogram (ECG). If any abnormalities are identified, an alternative medication such as memantine (a glutamate receptor stabiliser recommended in moderate to severe Alzheimer-type dementia) may be prescribed instead.^2^

Currently there are no specific national guidelines for clinicians to determine what cardiac investigations are appropriate prior to the commencement of AChEIs to screen for pro-arrhythmic potential. A published protocol and some regional and local guidelines exist to help guide practice,^10^ but there is no national consensus: some recommend that ECGs are performed only on high-risk patients, others perform ECGs on all patients. Anecdotal and regional evidence suggests that this has led to a wide variation in practice.^11^

This has potential practical and safety implications for the 331 memory clinics in the UK, the clinicians who work in them and the patients they treat. Beyond the important question of deciding which patients should have an ECG, clinics may have limited access to ECG machines or clinical rooms for performing ECGs. Furthermore, many lack the staff with the time or skills to perform the ECG. When ECGs cannot be performed in-house they are outsourced to other medical centres, such as general practices or local hospitals, which has both cost and practical implications to clinical services as well as patients. Once an ECG is obtained, a clinician with the necessary and up-to-date training needs time to interpret and process the results, before communicating a treatment decision.

It is not known how widely practice differs throughout the UK. Inconsistencies may have potential patient, organisational and economic implications.

## Aims

To understand and document what cardiac monitoring is routinely conducted in UK memory clinics before prescribing AChEIs, how investigations are organised and funded and how results are interpreted.

## Method

### Overview

We conducted a national web-based survey open to all UK-based clinicians working in memory clinics.

### Design

The survey was designed in collaboration with the Executive Committee of the Royal College of Psychiatrists’ (RCPsych's) Faculty of Old Age Psychiatry. It was conducted electronically via the website SurveyMonkey™ and was only open to clinicians (including doctors, nurses, psychologists and occupational therapists) actively working in the 331 memory clinics around the UK. Memory clinics were defined as a service that assesses, diagnoses and treats dementia as set out by the UK Memory Services National Accreditation Programme (MSNAP).^12^

The survey questions covered five main domains:

- basic demographic information about the participant, including where they worked (organised by Health Education England regional boundaries)^13^ and their role
- the current practice for conducting ECGs in their memory clinic, including whether this test was performed on all or selected patients before prescribing AChEIs
- the current governance, funding and organisational procedures for obtaining ECGs in their memory clinic
- the perceived service level advantages and disadvantages of obtaining ECGs from a local memory clinic
- the level of confidence clinicians have in analysing ECGs, as well as whether they have access to training programmes to keep their skills up to date.

### Survey distribution

A link to the survey was distributed by email, which also contained a description of the survey and its aims. To target the greatest possible number of healthcare professionals, two national distribution lists were used:

- the RCPsych's MSNAP distribution list (*n* = 331 memory clinics)
- the RCPsych's Faculty of Old Age Psychiatry membership list, which has a total 3685 members, some of whom (but not all) work in memory clinics.

A reminder email was sent to the same cohort after 2 weeks. The survey was closed after 8 weeks (when no new responses had been received for 2 weeks). Participants could only complete the survey once.

### Analysis

Descriptive statistics were used to display the collated responses.

### Ethics and information governance

All data requested were fully anonymised. Health Research Authority and ethics committee approval were not required.

## Results

### Demographics

A total of 305 healthcare professionals working in UK memory clinics responded to the survey. There was representation from all geographical regions of the UK, with the greatest number of respondents working in the North East and North West of England. The majority of respondents were consultant grade doctors (59%) and specialty grade doctors (13%) based in memory clinics (Table 1).

**Table 1:** Demographic information regarding respondents (*n* = 305)

	*n* (%)
Healthcare region	
Yorkshire & Humber	36 (11.8)
North West	33 (10.8)
West Midlands	29 (9.7)
Scotland	26 (8.7)
East of England	22 (7.3)
South West	21 (6.9)
Wales	19 (6.3)
North, Central and East London	17 (5.7)
Northern Ireland	16 (5.3)
East Midlands	16 (5.3)
Kent, Surrey and Sussex	15 (4.9)
South London	13 (4.3)
Thames Valley	12 (3.9)
Wessex	12 (3.9)
North East & Cumbria	11 (3.7)
North West London	2 (0.7)
Skipped question	5 (1.7)
Position	
Consultant psychiatrist	176 (58.7)
Specialty doctor	41 (13.7)
Nurse	32 (10.7)
Higher trainee in psychiatry	25 (8.3)
Core trainee in psychiatry	12 (3.9)
Consultant geriatrician	3 (1.0)
Consultant nurse	2 (0.7)
Memory services manager	2 (0.7)
Clinical psychologist	2 (0.7)
Healthcare assistant	2 (0.7)
Occupational therapist	2 (0.7)
Consultant neurologist	1 (0.3)
Skipped question	3 (1.0)

### Cardiac investigations before indicated AChEI

Of the 305 respondents, 113 (37%) were aware of the presence of local clinical guidelines that stipulated what cardiac monitoring should be conducted prior to commencing AChEIs; 121 (40%) respondents did not think that there was a guideline and 67 (22%) were unsure. Four respondents did not answer this question.

**Fig. 1:**
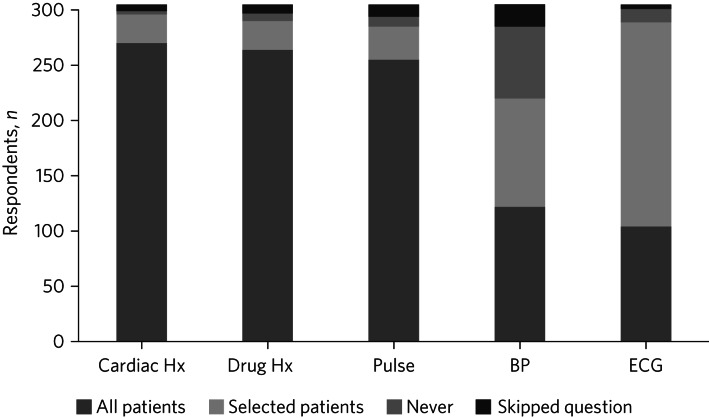
Cardiac monitoring reported by 305 UK memory clinic practitioners before prescribing acetylcholinesterase inhibitors. Hx, history; BP, blood pressure; ECG, electrocardiogram.

In total, 270 respondents (89% of 305) reported taking a cardiac history on all patients before prescribing AChEIs, 87% took a cardiac drug history and 84% took a pulse check. Fewer checked blood pressure as a standard (40%). Only 34% (*n* = 104) of respondents conducted an ECG on every patient before prescribing; 61% (*n* = 185) conducted ECGs only on selected patients and 4% never conducted an ECG (Fig. 1).

Regardless of whether or not they conducted an ECG on all patients, 41% (*n* = 124) of respondents were of the opinion that obtaining an ECG on every patient was clinically useful. Of respondents who conducted an ECG on all patients, 74% (*n* = 77 of 104) believed that obtaining an ECG was clinically useful, 23% (*n* = 24) believed that the practice was not clinically useful and 3 respondents did not answer this question. Of respondents who only conducted ECGs on selected patients, 69% (*n* = 127 of 185) were of the opinion that their practice was correct, 23% (*n* = 42) believed it was clinically useful to obtain an ECG on all patients and 16 respondents did not answer this question. Of those who indicated that they never perform an ECG before prescribing AChEIs (*n* = 12), 4 were of the opinion that an ECG was indicated in all patients and the remaining 8 were of the opinion that it was not.

### Service organisation

Of the 104 respondents who conducted ECGs on all patients, 45 (43%) had a service-level agreement in place stipulating which service would fund and perform the ECG; 24 (23%) respondents did not believe such an agreement was in place and 36 (35%) were unsure. In services that conducted ECGs on selected patients (*n* = 185), only 46 (25%) were aware of a service level agreement, 74 (40%) did not believe an agreement was in place and 50 (27%) were unsure. The remaining 14 participants did not answer this question.

Where respondents (*n* = 185) reported that their memory clinic conducted ECGs on selected patients, 12% of ECGs were conducted by memory clinic staff and 16% of ECGs were funded by the requesting memory clinic. The remaining ECGs were outsourced to and funded by either the local general practice or hospital, or the respondent was unsure how the ECG was obtained or funded at all. Where ECGs were conducted on all patients attending the memory clinic (*n* = 104 respondents), 29% were conducted by the memory clinic and 46% were funded by the memory clinic, with the majority being conducted and funded by the patients’ own general practice or local hospital (Table 2).

**Table 2:** Electrocardiogram (ECG) provision and funding reported for UK memory clinics that performed ECGs on selected patients compared with those that performed ECGs on all patients before prescribing acetylcholinesterase inhibitors

	Performed on selected patients (185 respondents)				Performed on all patients (104 respondents)			
	ECGs conducted		Funding		ECGs conducted		Funding	
	*n*	%	*n*	%	*n*	%	*n*	%
In-house	23	12	30	16	30	29	48	46
General practice	75	41	41	22	34	33	33	32
Hospital	37	20	7	4	22	21	10	10
General practice or hospital	26	14	0	0	15	14	0	0
Unsure	4	2	86	47	0	0	13	13
Other	6	3	6	3	3	3	0	0
Skipped question	14	8	15	8	0	0	0	0

### Patient impact

In the group who conducted ECGs on selected patients, 162 (88%) noted that obtaining an ECG caused the patients inconvenience, including:

- the need for an extra appointment with the patient's general practice (obtaining the ECG) and memory clinic (interpreting the ECG)
- additional travel to obtain the ECG
- AChEI treatment delays of 2–4 weeks (median) while the ECG was conducted and interpreted.

In the group who conducted ECGs on all patients, 77 (74%) reported that obtaining an ECG caused patient disruption. The most common reasons identified were extra appointments generated with general practices (*n* = 61) and memory clinics (*n* = 19). Excess patient travel was identified as a barrier for obtaining an ECG by 27 respondents.

### ECG interpretation

**Fig. 2:**
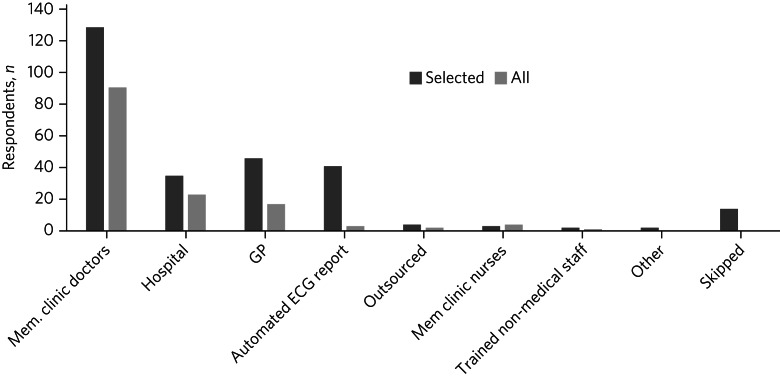
Electrocardiogram (ECG) interpretation reported by 270 UK memory clinic practitioners for clinics that conducted ECGs on all patients before prescribing acetylcholinesterase inhibitors. Mem., memory; GP, general practice.

The majority of respondents reported that ECGs were interpreted by clinicians working in memory clinics. In a small number of cases, the ECGs were sent to the local cardiology department or general practice. Automated ECG analysis was used for interpretation in a number of centres. In clinics that conducted ECGs on all patients, it was more common for ECGs to be interpreted in the memory clinic (Fig. 2).

Of 244 respondents who indicated they interpret ECGs in the memory clinic, 8 received mandatory regular ECG interpretation training updates at least every 5 years and a further 74 organised their own training. The confidence that healthcare professionals tasked with interpreting ECGs had in their ability to do so was variable. Of the 244 respondents, 8% were very comfortable, 36% fairly comfortable, 30% neither comfortable or uncomfortable, 17% fairly uncomfortable and 10% indicated that they would not attempt to interpret results.

## Discussion

### Meaning of the study

AChEIs are a class of drug that offers modest patient benefits in Alzheimer-type dementia.^14^ Patients expect safe prescribing practice, and there is an imperative for the prescriber to act in their best interests. This is perhaps particularly pertinent in memory clinics, where some people with dementia might lack capacity to make decisions about whether to take a drug or not.^15^ The General Medical Council ethical guidance on prescribing advises that a doctor ‘must only prescribe a drug when they have adequate knowledge of a person's health’.^16^ Cardiac assessment before prescribing AChEIs, including a pulse, cardiac history or an ECG, could be considered part of this acquisition of knowledge. This survey has brought to light inconsistencies in gathering this knowledge that have potential clinical, economic and patient implications.

### Clinical practice

It is clear from the results that there are considerable differences in opinion and clinical practice as to what cardiac monitoring should be done before prescribing AChEIs. The majority (>85%) carry out a simple history and pulse check. However, the question as to whether an ECG should be done for all, selected or no patients divides opinion and practice. The stark differences observed suggest that one group is engaging in either over- or under-cautious prescribing practice. This survey is unable to answer the question of which practice is safest. Evidence published to date does suggest a low rate of cardiac side-effects,^17,18^ and strong arguments have been made that checking pulse only may suffice in the majority of cases.^10^ However, this does not appear to have unified practice.

One must also consider the impact of conducting ECGs and discovering incidental findings that are of questionable clinical significance. This could lead to increased patient anxiety and unnecessary follow-up appointments with specialists.

### Organisational practice

Regardless of whether respondents performed ECGs on all or selected patients before prescribing AChEIs, the majority were not able to provide the ECG in-house.

This suggests that the majority of patients have to book a separate appointment at, and arrange travel to, their general practice or local hospital to obtain an ECG. A further appointment at the memory clinic is then arranged, where the ECG is interpreted and medications prescribed. This can cause distress in patients who have dementia, and 84% of respondents indicated that it also caused treatment delays.

The average cost of conducting an ECG is £45, with the additional cost of the ECG appointment at a general practice or hospital and a follow-up appointment in the memory clinic. In the few clinics that do ECGs in-house there are still up-front costs: the ECG machine and consumables (electrodes and paper), staff time and estate space. The majority of respondents either did not fund outsourced ECGs or were unsure who did. Outsourcing cost has large economic and organisational implications for the provider and can cause tension between services if it is not made explicit who is responsible for the test.

ECG interpretation can be challenging and complex. Apart from physicians such as cardiologists and internal medicine specialists, who are acquiring and interpreting ECGs on a daily basis, healthcare professionals who interpret ECGs infrequently or *ad hoc* may not be as confident or sufficiently qualified to interpret them.^19^ It is therefore important that regular training sessions are in place for practitioners to keep their knowledge up to date to ensure patient safety. The survey suggests that in the majority of cases this is not happening.

### Strengths and weaknesses

We believe that this is the first national study of its kind to be conducted in UK memory services. The number of responses received (305) was reasonable and data reflects that of smaller regional surveys, suggesting reliability.^11^

No sampling frame was used, and individual memory clinics were deliberately not targeted as it was believed that this might create a social desirability response bias. Instead, the invitation to participate was sent to wide group mailing lists (the RCPsych's MSNAP distribution list and Old Age Faculty's membership list). These groups are highly likely to contain memory clinic practitioners but are not exclusive to them. Consequently, the number of people the survey was distributed to was large (331 and 3685 respectively), but only a proportion of them would have been eligible to complete it. This creates an impression of a low response rate (*n* = 305, 8%), but the true response rate is unknown. This limits the generalisability of the data. The advantage of this technique was that the survey had good potential to reach its intended audience.

Participant self-selection potentially introducing bias, with individuals taking the survey to validate practice they are unsure of or believe they are doing well. This further limits the generalisability of the results.

A further potential area of variation in clinical practice is what ECG changes would contraindicate the prescribing of an AChEI. This was not explored in the survey and is potential area of future work.

### Implications for clinical practice

We believe the results of this survey indicate the need for an evidence-based review of the cardiac safety of AChEIs and a nationally led guide to best practice. Without this, patients are potentially at risk of harm and healthcare organisations are not operating efficiently. Although some guidance has been suggested in the academic literature, for example Rowland et al's algorithm,^10^ our results suggest that it has not united opinion or practice.

It is likely that whatever future prescribing guidance is available, some patients will still require an ECG and for the foreseeable future practice is unlikely to change. Local service-level agreements as to how the ECG is provided and funded may help reduce confusion and tension between different NHS departments. Alternatively, other solutions for providing ECGs safely in memory clinics at the time of the appointment may be considered. We think that hand-held app-based ECG technology may have a role in allowing memory clinics to conduct ECGs rapidly and initiate treatment without delays. However, the ability of these devices to sensitively measure pulse rate and QTc interval is so far unknown. We are currently conducting trials to evaluate this.

Ensuring consistently safe interpretation of ECGs is imperative. Organising regular training for memory clinic ECG analysis could improve patient safety and well-being and prescribers’ confidence. As part of national guidance on ECG provision, mandatory regular training could be considered.

## About the authors

**George Crowther**, MBBS, BSc, MSc, DGM, MRCPsych, PhD, Consultant Old Age Psychiatrist, Department of Liaison Psychiatry, Leeds and York Partnership NHS Foundation Trust, Leeds, UK; and visiting Senior Lecturer, Academic Unit of Psychiatry and Behavioural Sciences, University of Leeds, UK; **Noura Ahmed**, MBBS, MRCP, Research Fellow, Department of Cardiology, Leeds Teaching Hospitals NHS Trust, UK; **Deepa Kasa**, MBBS, MRCPsych, Higher Trainee in Psychiatry, Leeds and York Partnership NHS Foundation Trust, Leeds, UK; **Zoe Goff**, MBChB, Higher Trainee in Psychiatry, Leeds and York Partnership NHS Foundation Trust, Leeds, UK; Muzahir H. Tayebjee, MBChB, MRCP, MD, Consultant Cardiac Electrophysiologist Department of Cardiology, Leeds Teaching Hospitals NHS Trust, UK.

## Data availability

The data that support the findings of this study are available from the corresponding author on reasonable request.
